# The Ebola crisis and people with disabilities’ access to healthcare and government services in Liberia

**DOI:** 10.1186/s12939-021-01580-6

**Published:** 2021-11-24

**Authors:** Maria Kett, Ellie Cole, Lucila Beato, Mark Carew, Richard Ngafuan, Sekkoh Konneh, Tim Colbourn

**Affiliations:** 1 UCL Institute of Epidemiology and Healthcare, London, UK; 2 UCL Institute of Epidemiology and Healthcare, London, UK; 3 University of Witwatersrand, Johannesburg, South Africa; 4 Leonard Cheshire, London, UK; 5 University of Liberia, Monrovia, Liberia; 6 UCL Institute for Global Health, London, UK

**Keywords:** Ebola, people with disabilities, Liberia, infectious diseases, inclusion

## Abstract

**Background:**

There has been little research on the impact of the 2014-2015 West African Ebola crisis on people with disabilities. This paper outlines the way in which the Ebola Virus Disease (EVD) outbreak in Liberia in 2015 highlighted existing inequalities and exclusion of people with disabilities and their households.

**Methods:**

The results presented here are part of a larger ESRC/DFID-funded mixed methods research project in Liberia (2014-2017) which included a quantitative household survey undertaken in five counties, complemented by qualitative focus group discussions and interviews with people with disabilities and other key stakeholders. Uniquely, this research gathered information about people with disabilities’ experience of the EVD outbreak, as well as additional socioeconomic and inclusion data, that compared their experience with non-disabled community members.

**Results:**

Reflections by people with disabilities themselves show knowledge, preparation, and responses to the EVD epidemic was often markedly different among people with disabilities due to limited resources, lack of inclusion by many mainstream public health and medical interventions and pre-existing discrimination, marginalisation and exclusion. Interviews with other key stakeholder revealed a lack of awareness of disability issues or sufficient training to include this population systematically in both Ebola response activities and general health services.

Key findings include the need to understand and mitigate direct and indirect health consequences of unequal responses to the epidemic, as well as the limited capacity of healthcare and social services to respond to people with disabilities.

**Conclusion:**

There are lessons to be learned from Ebola outbreak around inclusion of people with disabilities, relevant to the current COVID-19 pandemic. Now is the time to undertake measures to ensure that people with disabilities do not continue to be marginalised and excluded during global public health emergencies.

**Supplementary Information:**

The online version contains supplementary material available at 10.1186/s12939-021-01580-6.

## Introduction

### Background

In 2014-2015, along with Sierra Leone and Guinea, Liberia experienced the worst ever outbreak of human Ebola Virus Disease (EVD) with over 28,000 cases, and 11,000 reported deaths directly attributed to Ebola across the three countries, and many communities quarantined or otherwise adversely affected economic and socially [5]. It has been argued that weak health systems – and weak political systems – exacerbated the spread of Ebola, and in Liberia specifically this probably contributed more so than funeral or other cultural practices – compounded by what Farmer calls the lack of “ …staff, the stuff or the space.” ([12]:157). Others have argued that chronic underinvestment bolstered by multi-lateral aid that does not improve core health systems paved the way for the Ebola disaster to unfold the way it did [4]. Leach [20] argues that “…an interlocking of institutions has contributed to longer-term, and interlaced, inequalities, unsustainabilities and insecurities…” (ibid: 820). This distrust, as evidenced during the West Africa Ebola crisis, can lead to suspicion about activities and interventions that ostensibly do have public good at their heart ([20]: 827).

Wilkinson and Fairhead [32] paint a nuanced picture of the relationship between historical contexts and contemporary trust in responses to Ebola in Sierra Leone and Guinea; in Liberia, Blair et al. [5] found that Liberians who expressed low trust in government were much less likely to comply with EVD control measures or policies, but found no evidence to suggest they were any more or less likely to hold ‘erroneous beliefs’ about EVD transmission, symptoms and treatments which they interpret to indicate a “…lack of trust the capacity or integrity of government institutions to promote mechanisms and implement policies to slow EVD's spread…” ([5]: 90). Only by shifting towards a more engaged approached with trusted members of the communities (e.g., religious leaders), relaxing draconian burial regulations and working with communities, did the state succeed in controlling the epidemic. Many researchers have argued that the communities themselves largely facilitated this, undertaking their own quarantining and monitoring [1, 26, 28].

Unfortunately, one important component of the impact of the Ebola outbreak was consistently overlooked. This was the link between Ebola and disability – with two different but interrelated issues: the impact of the disease on people with pre-existing disabilities; and the disabling consequences of the disease itself ([4]. According to one study from Sierra Leone, EVD survivors are up to seven times more likely to have long-term impairments including ocular, musculoskeletal, and neuropsychiatric sequelae [14].

People with disabilities may be at more risk of contracting Ebola (or indeed other highly infectious diseases including COVID-19), given that many are poorer, live in substandard accommodation, have precarious employment or income, and may rely on others to provide support or care, as well as having underlying health conditions that place them at higher risk of becoming more seriously ill if infected [23]. However, it is equally clear that as a group, the rights and needs of people with disabilities are rarely considered a priority when planning interventions, despite a range of international commitments, including the UN Convention of the Rights of Persons with Disabilities (UNCRPD; UN [30]), which Liberia signed in 2007 and ratified in 2012.; the UN Disability Inclusion Strategy [31], and IASC Guidelines on the Inclusion of Persons with Disabilities in Humanitarian Emergencies (2019).

However, while focusing on the disabling consequences of Ebola is important, widening the focus further to examine how persons with disabilities, their families, and their caregivers were included in the Ebola response, and how Ebola affected their lives, is an important component of understanding wider issues around equity and social justice for marginalised and excluded groups. To date there has been little research on the impact of the Ebola crisis on people with disabilities, nor how (mis)trust plays out in these experiences. In this paper we seek to fill this gap through an analysis of the experiences of people with disabilities during the outbreak in Liberia of key areas of healthcare access, participation in community and political life and other indicators of subjective and objective wellbeing, stratified by their community’s exposure to Ebola.

### Methods

This work is part of a larger study investigating multidimensional poverty at individual and household levels and the political and institutional conditions needed for poverty reduction for people with disabilities in Liberia [8, 16].

The original research did not set out to focus on Ebola, but the outbreak began during the initial phase of the project, affording the unique opportunity for research on the consequences and impacts of the outbreak on people with disabilities in comparison to their non-disabled counterparts. In response, a set of Ebola-related questions was added to a household survey tool [8]. Research on other infectious diseases (in particular HIV/AIDS) has demonstrated how persons with disabilities are at significant disadvantage compared to their non-disabled counterparts for a range of reasons, including multidimensional poverty, gender, risk of sexual violence and limited social networks [10]

In this paper we focus on findings from these Ebola questions including those on decision-making, health-seeking behaviours, and the impact of the crisis on individual, household and community life.

Findings from the comparative household survey of disabled and non-disabled people have previously been published [8]. This study was conducted between February and May 2016 in five counties in Liberia: Cape Mount, Lofa, Grand Bassa, Monserrado, and Sinoe (not all areas were directly affected by Ebola, for example, Ebola was reported only in parts of Grand Bassa). The survey was designed to capture subjective and objective wellbeing in multiple domains, drawing on indicators used by the authors in previous studies, and incorporated the Cummins et al [9] Personal Wellbeing Index.

Sampling involved the selection of two types of households: households that included a person with disabilities; and a non-disabled household in the same community. To identify households with a person with disabilities, a list of contacts was provided by the National Union of Persons with Disabilities (NUOD) and a random sampling of this list was undertaken to select respondent households. To select a non-disabled household for comparison, the next nearest household was approached. For both types of households, the household head was surveyed, and within disabled households, the person with disabilities also completed the survey, as well as an additional household member who was randomly selected from the household list. In the non-disabled household, a household member was also surveyed who most closely matched the neighbouring person with disabilities by age and sex. The approach was utilised to allow intra- and inter-household comparison of dimensions of wellbeing . The survey was conducted in 485 disabled and 538 non-disabled households (992 and 1028 respondents respectively, total 2,020 individuals).

In addition to the survey, the team also undertook 34 key informant interviews with community leaders (including village chiefs and traditional healers), government officials, healthcare professionals and community health volunteers, and other stakeholders in the same five counties. Key informants were identified with the support of NUOD and other members of the research team. The team also undertook 22 focus group discussions (FGDs) with men and women with disabilities, plus six FGDs with Ebola survivors, with a total of 106 male and 98 female participants (with around five to ten people per group). Participants for the focus groups were identified by NUOD and Ebola survivor groups. Qualitative research activities were undertaken in all five of the focus counties.

Discussion guidelines were developed for the FGDs, and semi-structured interview (SSI) questionnaires were developed for healthcare workers, community leaders and traditional healers. Both the SSIs and FGDs focused on themes covered in the household survey (health, education, livelihoods, transport, community, crime and safety, and political engagement) but explored them in more detail, particularly around the impact of Ebola on communities. MK and EC undertook the training of the research team (which included colleagues from NUOD). Interviews and focus groups were undertaken in Liberian-English and were recorded and notarised. Following completion of the fieldwork, all data was transcribed by the research team in Liberia. All transcripts were shared with the UK research team and an initial manual sift of themes was undertaken. EC undertook manual coding of initial theme content analysis. There were then agreed with team and structured into preliminary findings [6] . Preliminary findings were presented for validation to community and organisational representatives in Monrovia, including the Disability Alliance during the final year of the research.

### Analysis

In this paper we bring together the findings from the household survey, as well as qualitative interviews, to provide a comprehensive overview of how people with disabilities fared in the face of the Ebola epidemic.

The survey results were analysed by disaggregating responses from disabled and non-disabled households for the following categories: Ebola-exposed households, non-exposed and unsure of exposure. Where multiple responses were possible (see Questionnaire: Appendix 1) we tabulate responses given by more than 10% of respondents.

The odds of the tabulated responses (outcomes) were compared in each of the respondent categories (disabled, non-disabled households in Ebola-exposed, unexposed and unsure of exposure) compared to the reference group of non-disabled households in unexposed areas, in separate multi-level mixed effects logistic regression models adjusting for age, sex, education, wealth quintile and clustering by village. Thirty-seven outcomes were thus compared, each with five comparisons to a reference group (i.e., 185 comparisons). We adjusted for multiple hypothesis testing via the Benjamini and Hochberg false discovery rate [3], which adjusts the p-value based on the number of comparisons made so that thresholds of statistical significance equivalent to single hypothesis testing can be given for each comparison. To ensure that the tabulated results are intuitive, only significant findings of *p*<0.05, *p*<0.01 and *p*<0.001, and p<0.0001 are highlighted following these adjustments.

## Results

Based on the interviews and survey findings, a number of key barriers can be identified. These are: the restrictions on community life; healthcare access; socioeconomic impacts; inclusion and access to information and behaviours and attitudes. We discuss each of these in turn below.

### Restrictions on movement and socialising

*“The Ebola news spread like when war is coming, everyone was discussing it in the community, at the market and in every street corner of this city. Friends and other people were coming in the community telling us the sickness is bad so don’t touch dead body, don’t accept stranger in your house and do not visit anybody. The health workers and other people in the community here were going around giving the Ebola information.” (Female with disabilities, Grand Bassa)*

This quote illustrates how quickly the news spread about Ebola outbreaks in communities. According to the survey data, 560 respondents reported some or many cases of Ebola in their communities; these were spread across disabled and non-disabled households and concentrated in Lofa, Cape Mount, and Montserrado (Table 1), some of the most affected counties in Liberia [19]. Overall, 460 people reported their household or community was affected by quarantining, with the majority reporting some or many cases in their communities.

**Table 1:** household reported being affected by Ebola in survey counties

	A few, or many, cases of Ebola (*n*=560)			No Ebola cases (*n*=1343)			Don’t know whether there were any Ebola cases (*n*=107)		
	Disabled household n (%)	Non-disabled household n (%)	Total n (%)	Disabled household n (%)	Non-disabled household n (%)	Total n (%)	Disabled household n (%)	Non-disabled household n (%)	Total n (%)
**County**									
Grand Bassa	14 (64%)	8 (36%)	22 (100%)	133 (47%)	147 (53%)	280 (100%)	3 (30%)	7 (70%)	10 (100%)
Cape Mount	59 (48%)	64 (52%)	123 (100%)	104 (43%)	140 (57%)	244 (100%)	21 (75%)	7 (25%)	28 (100%)
Lofa	148 (48%)	161 (52%)	309 (100%)	103 (61%)	65 (39%)	168 (100%)	1 (7%)	13 (93%)	14 (100%)
Montserrado	42 (42%)	59 (58%)	101 (100%)	106 (48%)	116 (52%)	222 (100%)	14 (47%)	16 (53%)	30 (100%)
Sinoe	4 (80%)	1 (20%)	5 (100%)	229 (53%)	200 (47%)	429 (100%)	4 (16%)	21 (84%)	25 (100%)
**Total**	**267 (48%)**	**293 (52%)**	**560 (100%)**	**675 (50%)**	**668 (50%)**	**1343 (100%)**	**43 (40%)**	**64 (60%)**	**107 (100%)**

A total of 1808 out of 2020 respondents reported being affected by Ebola. The most reported impacts were a decreased community social life, stopping welcoming visitors and strangers, stopping communal eating, and restricted movement in and out of the community (Table 2). Those in disabled households in Ebola-exposed areas were much more likely to report decreased social life compared with non-disabled households or in areas unexposed, or with unknown exposure, to Ebola, and this finding was highly statistically significant after controlling for age, sex, education, wealth and village-level clustering (fully adjusted models, Table 2).

**Table 2:** How did Ebola affect you and your community

	A few, or many, cases of Ebola (*n*=560)			No Ebola cases (*n*=1343)			Don’t know whether there were any Ebola cases (*n*=107)		
	Disabled household n (%)	Non-disabled household n (%)	Total n (%)	Disabled household n (%)	Non-disabled household n (% (reference group)	Total n (%)	Disabled household n (%)	Non-disabled household n (%)	Total n (%)
***How did Ebola affect you and your community?***^*1*^									
Decreased social life	211 (80%)**	90 (31%)	301 (54%)	261 (42%)	218 (38%)	479 (40%)	7 (29%)	11 (35%)	18 (32%)
Stopping welcoming visitors and strangers	19 (7%)	50 (17%)**	69 (12%)	111 (18%)**	53 (9%)	164 (14%)	8 (33%)	2 (6%)	10 (18%)
Stopping communal eating	24 (9%)	21 (7%)††	45 (8%)	94 (17%)	85 (14%)	179 (15%)	1 (4%)	7 (23%)	8 (15%)
Restricted movement	4 (2%)†	63 (22%)**	67 (12%)	67 (11%)	80 (14%)	147 (12%)	7 (29%)	1 (3%)	8 (15%)
Close down of schools	3 (1%)††	25 (9%)††	28 (5%)	34 (5%)††	91 (16%)	125 (10%)	0 (0%)	6 (19%)	6 (11%)

** *p*<0.0005 *p<0.005 significant increase in odds †† *p*<0.0005 †*p*<0.005 decrease in odds of outcome compared to reference group of Non-disabled households in no Ebola cases area, in multi-level mixed effects logistic regression adjusting for age, sex, education, and wealth quintile and clustering by village

For some people with disabilities, reduction in social life was intentional, a response to public health advice, such as ‘remain at home’ and ‘avoid contact with others’:

*“We were careful with ourselves because we disabled people were not going in the streets, because we were not to touch people - even in the car it became a problem”* (Male respondent with disabilities Bonard Farm, Montserrado).

This point is important, as many people with disabilities reported relying on street begging to top up incomes or pay for additional – and usually unexpected – expenses, such as healthcare:

“*When I get sick I can go in the street and beg and when I get any money I go to the drug store and buy medicine and take it. But I don’t go to hospital because I don’t have the money to go there*.” (Female with disabilities, Bonard Farm Community, Montserrado)

Disabled households in Ebola-exposed and unexposed areas were more likely to report they stopped welcoming visitors or strangers. However, they did not report significantly different levels of stopping communal eating, or restricted movement, compared to non-disabled households in Ebola-unexposed areas (Table 2). Indeed, disabled households in Ebola-exposed areas were less likely to report restricted movement than non-disabled households in Ebola unexposed areas (Table 2). One reason may have been the need to supplement incomes through begging. The impact of restricted movement was felt keenly by those who relied on begging:

*“…if we don’t beg, how we will eat, how will we support the children?”* (Male with disabilities, Fiamah Community)

Therefore, while at least some non-disabled households could reduce outside contact, disabled households were often unable to do so because of their reliance on street begging. If people with disabilities did restrict their movements, then they became more reliant on others:

*“…for me, I can’t go in the street to beg, I can call people and ask them to help me, that’s how my family and I are surviving”* (Female with disabilities, Bonard farm community)

### Healthcare access

Households with and without people with disabilities in Ebola-exposed areas, as well as disabled households in areas without Ebola cases, were significantly more likely to report access to health services worsened compared to non-disabled people in areas without Ebola (Table 3, fully adjusted models). Disabled households in Ebola-unsure areas and non-disabled households in Ebola-exposed were significantly less likely to get treatment in health facilities during the Ebola outbreak than non-disabled people in areas without Ebola cases (Table 4, fully adjusted models).

**Table 3:** Access to health service

	A few, or many, cases of Ebola (*n*=560)			No Ebola cases (*n*=1343)			Don’t know whether there were any Ebola cases (*n*=107)		
	Disabled household n (%)	Non-disabled household n (%)	Total n (%)	Disabled household n (%)	Non-disabled household n (%) (reference group)	Total n (%)	Disabled household n (%)	Non-disabled household n (%)	Total n (%)
***During the Ebola outbreak did your access to health services:***									
Get better	2 (1%)	7 (2%)	9 (2%)	18 (3%)	30 (5%)	48 (4%)	1 (2%)	1 (2%)	2 (2%)
Stay the same	45 (17%) ††	57 (20%) ††	102 (18%)	131 (20%) ††	298 (46%)	429 (33%)	17 (40%)	22 (40%)	39 (40%)
Get worse	217 (82%)**	217 (75%)**	434 (78%)	458 (69%)**	308 (47%)	766 (58%)	14 (33%)	20 (36%)	34 (35%)
Don't know	1 (0%)	9 (3%)	10 (2%)	59 (9%)**	17 (3%)	76 (6%)	10 (24%)**	12 (22%)**	22 (23%)

** *p*<0.0005 *p<0.005 significant increase in odds †† *p*<0.0005 †*p*<0.005 decrease in odds of outcome compared to reference group of Non-disabled households in no Ebola cases area, in multi-level mixed effects logistic regression adjusting for age, sex, education, and wealth quintile and clustering by village

**Table 4:** Treatment during the Ebola outbreak

	A few, or many, cases of Ebola (*n*=560)			No Ebola cases (*n*=1343)			Don’t know whether there were any Ebola cases (*n*=107)		
	Disabled household n (%)	Non-disabled household n (%)	Total n (%)	Disabled household n (%)	Non-disabled household n (%) (reference group)	Total n (%)	Disabled household n (%)	Non-disabled household n (%)	Total n (%)
***How did you get treatment during the Ebola outbreak?***									
Did not need treatment	9 (3%) ††	88 (31%)**	97 (18%)	99 (15%)	124 (21%)	233 (18%)	3 (7%)	10 (18%)	13 (13%)
Health facilities	20 (8%)	23 (8%) ††	43 (8%)	77 (12%)	129 (20%)	206 (16%)	18 (42%) ††	7 (12%)	25 (25%)
Traditional healers	49 (18%)**	15 (5%)	64 (12%)	47 (7%)*	20 (3%)	67 (5%)	2 (5%)	2 (4%)	4 (4%)
Self-treatment (including pharmacy)/treatment from household	181 (68%)**	146 (51%)	327 (60%)	418 (63%)**	328 (51%)	746 (57%)	10 (23%) †	22 (39%)	32 (32%)

** *p*<0.0005 *p<0.005 significant increase in odds †† *p*<0.0005 †*p*<0.005 decrease in odds of outcome compared to reference group of Non-disabled households in no Ebola cases area, in multi-level mixed effects logistic regression adjusting for age, sex, education, and wealth quintile and clustering by village

Many people actively avoided going to a hospital during the outbreak, including people with disabilities:

*“During the Ebola outbreak I did not go to any hospital for treatment because I was afraid of the Ebola. At that time when you are suffering from headache or malaria and you to the hospital the doctors will keep you there for long time saying that they are observing you. That is what everybody was afraid to go to the hospital. When I heard the news that a sickness in Monrovia call Ebola and it is spreading, I went quickly to the drug store with the little money I have and buy drugs for me and my children.”* (Female respondent with disabilities, Buchanan, Grand Bassa)

Disabled households in both Ebola-exposed and -unexposed areas were significantly more likely to report getting treatment from traditional healers or self-treating compared with non-disabled people in unexposed areas (Table 4). For some people, this was due to fear about going to a health centre:

*“For me I was afraid to go the hospital so I used to buy my medicines from the guys who can sell medicines around…the medicines help me because I did not come down with any major sickness [needing] to go to the hospital so I did not go there during the Ebola.” (*Male with disability, Grand Bassa)

Other people with disabilities prepared by stockpiling medicines they might need or used whatever they had, despite possible complications with self-medication. In Ebola affected areas, both disabled and non-disabled females were also more likely to report self-medicating compared to males.

Many health professionals interviewed reported being overwhelmed by EVD, by the measures required to ensure everyone in the community was aware of the public health messages and by the stringent guidelines to follow if they suspected someone had Ebola. Many also reported already well-documented responses, such as communities hiding sick people, or infected persons running away to avoid hospitalisation [25].

Yet few healthcare workers acknowledged any challenges with the delivery of services for people with disabilities. None of the healthcare workers interviewed - from directors of hospitals down to General Community Health Volunteers (GCHVs) - had received any training around disability issues, despite the government’s commitments to the human rights of people with disabilities. GCHV are voluntary posts, monitoring disease outbreaks and providing advice to community members, encouraging them to go for tests and treatment as needed. All GCHVs interviewed reported having limited resources and covering large geographic areas. In these circumstances people with disabilities were not a priority. Only one nurse reported her hospital tried to ensure people with disabilities could access treatment, by sending the ambulance if they needed transport, and ensuring priority treatment on arrival to avoid long queues. Yet no practitioner mentioned anything about access to information, such as Sign Language interpreters. As one GCHV acknowledged, during the Ebola outbreak:

“…*some people did not receive proper health care, like you mentioned the disabled people we don’t even know how they were living, so the need of everyone was not addressed. Maybe they wanted something they were not getting …and we did not pay attention to that*.” (GCHV, Sinje, Grand Cape Mount)

Whilst healthcare workers acknowledged rarely seeing people with disabilities accessing clinics, they did not question why this might be. Inaccessibility of services due to cost, physical inaccessibility or discrimination were not considered. Unsurprisingly, when healthcare workers encountered adults and children with disabilities, some reported struggling to know how best to support them.

When Ebola struck, people with disabilities therefore decided on accessing treatment based on a range of factors: how unwell they were, fears about going to health centres where they might contract EVD, amount of money they had for care, and long-standing experience with inaccessible health facilities and staff that had little or no knowledge or training on disability.

## Socioeconomic impacts

“*The hardship was experienced at all levels in the county. All our development projects stop because of Ebola outbreak and the County Economy broke down. We are still experiencing the effect on our economy today.”* (County Engineer, Grand Bassa)

The county engineer quoted above raises an important point about the financial implications of the outbreak. The long-term impacts go beyond health. As the respondent notes, it caused chaos, and Liberia is still recovering. These included socioeconomic impacts, including reduced community cohesion, loss of education, reduced child protection, widespread job losses and food insecurity [11, 18]. People with disabilities were already struggling in all these areas before the outbreak [13]. After the outbreak, consequences continued. As one disabled woman noted:

“*I have two girl children and one of them finish with high school last year and the funding for her to go to college is what [is] giving me hard time. The other one pass to the twelve grade and because of the Ebola where the government close and open school for half semester. I was not able to send her to school this year so I asked her to go to trade school until I can get money then next year she will go back to school*.” (Female with disabilities, Bonard Farm Community)

It is unclear how long it will take this mother to earn enough money to send her daughter back to school. Thus, the impacts for this family are far reaching in terms of loss of potential earnings and opportunities.

Lack of government support was raised by several respondents. Despite a *National Social Protection Policy and Strategy for Liberia* (2013), which acknowledges that people with disabilities may be specifically vulnerable, there is very little in the way of provision of social protection, leaving many reliant on NGOs to provide goods and services. Few, if any, of the participants mentioned social protection specifically, though many highlighted the need for ‘support’ and ‘empowerment’ through the vocational training or other means of support. This consistent exclusion has led people with disabilities being overly reliant on charity, and the good will of benefactors, rather than raising questions about state responsibility to deliver on existing commitments for people with disabilities, including social protection:

“*One man…was the one who even help us and deliver our sister when she was in labour pain but all the hospital them around here refused her. I even used to go there for treatment to his small clinic.. …the man say his brother is blind so whenever he see blind people he help them and we can be paying the money small small until we complete the payment.*” (Female with disabilities, Bonard Farm Community, Montserrado)

### Inclusion and access to information

Lack of inclusion in community activities before the outbreak inevitably prompted discussion about the extent to which people with disabilities were included during the outbreak itself. For example, disability leaders spoke about challenges of disseminating information, such as during meetings:

“…*there were challenges because to bring a disabled person in meetings was little bit difficult in that they cannot move easily and you need transportation to do so, sometime these meeting are call to far distances and to move the disabled from one point to the other is not an easy task”* (Community Chairman; Buchanan District, Grand Bassa)

Others reflected on the assumption that people with disabilities were included as part of the general outreach efforts, rather than specifically targeting them to ensure inclusion, As one leader noted:

“…*yes and no: yes, in that since that we reached out to all of the communities and they are part of the community. No, in the way that we were not focusing on them specifically; so that is why I said yes and no*” County Inspector, Robertsport, Grand Cape Mount)

Other officials reported they targeted people with disabilities specifically, but that it was ‘time consuming’, and there were no funds allocated to cover this inclusion, resulting in ad hoc approaches and complaints from some people with disabilities that they were left out during the epidemic and in subsequent recovery efforts.

Inclusion during and after the epidemic also raises the question of where those in disabled households were getting their information from. Some reported getting it from the radio, though that would exclude those without radios, and those who were Deaf or hearing impaired. Some reported getting information from town criers, the Chief, or friends, sources which may lead to misinformation or misunderstanding. The majority of respondents listened to community leaders when making Ebola-related decisions (Table 5, fully adjusted models,). Respondents in disabled households were more likely to listen to community leaders than respondents in non-disabled households in both Ebola-exposed and unexposed areas (Table 5). By contrast, non-disabled households in Ebola-exposed areas were more likely to listen to government/local authority representatives than disabled households and those in unexposed or unknown exposure to Ebola areas; perhaps worryingly, respondents in disabled households in Ebola-exposed areas were less likely to listen to health workers than other groups of respondents (Table 5).

**Table 5:** Who did you and your community listen to when making decisions about how to stop Ebola?

	A few, or many, cases of Ebola (*n*=560)			No Ebola cases (*n*=1343)			Don’t know whether there were any Ebola cases (*n*=107)		
	Disabled household n (%)	Non-disabled household n (%)	Total n (%)	Disabled household n (%)	Non-disabled household n (%) (reference group)	Total n (%)	Disabled household n (%)	Non-disabled household n (%)	Total n (%)
***Who did you and your community listen to when making decisions about how to stop Ebola?*** ^1^									
Community leaders	240 (91%)**	107 (38%)††	347 (64%)	508 (80%)**	380 (69%)	888 (75%)	21 (78%)	19 (61%)††	40 (69%)
Government/local authority representatives	4 (2%)	112 (40%)**	116 (21%)	19 (3%)	17 (3%)	36 (3%)	2 (7%)	6 (19%)	8 (14%)
Health workers	10 (4%)††	28 (10%)	38 (7%)	74 (12%)	116 (21%)	190 (16%)	2 (7%)	3 (10%)	5 (9%)

** *p*<0.0005 *p<0.005 significant increase in odds †† *p*<0.0005 †*p*<0.005 decrease in odds of outcome compared to reference group of Non-disabled households in no Ebola cases area, in multi-level mixed effects logistic regression adjusting for age, sex, education, and wealth quintile and clustering by village

However, many got information from a myriad of sources, such as this woman:

*“I got the news from family, friends, and on radio, everybody was talking about this sickness how bad it is and quick to kill. So before the Ebola reach here people from the hospital and the community were coming and telling us about it. They told us not to shake hands, we should wash our hands all the time, we should not eat monkey and bat meat and we should not accept stranger[s] in our house even if the person is our family member”.* (Female with disabilities, Grand Bassa)

The lack of materials in alternative forms or languages is also an issue that came up during the FGDs, a particular concern for people with disabilities who use sign language or have less formal education:

*“Some of the boys from our town here were used to give the Ebola messages in our dialect to help those who cannot understand English to understand the messages.” (*Female, Jenneh Wende, Grand Cape Mount)

There were attempts to make public health information accessible to all, including wall murals graphically depicting how EVD spreads. The interviews and survey results show that people with disabilities received some information. However, the timeliness and accuracy of this information is unclear.

### Behaviour and attitudes

“…*during the Ebola the fear was too much - the way people were exaggerating things that time the fear was there*” (Male with disabilities, Robertsport, Grand Cape Mount)

The above quote illustrates how lack of information and misinformation made people afraid during the outbreak. This affected how people behaved towards each other, and towards EVD survivors. According to our survey, respondents reported that their community changed how they acted towards one another during the outbreak. Further, significantly higher proportions of both disabled and non-disabled respondents in Ebola-affected areas, and disabled respondents in non-affected and uncertain areas, reported changes in behaviour compared to non-disabled respondents in non-affected areas (Table 6, fully adjusted models).

**Table 6:** Attitudes and treatment during Ebola

	A few, or many, cases of Ebola (*n*=560)			No Ebola cases (*n*=1343)			Don’t know whether there were any Ebola cases (*n*=107)		
	Disabled household n (%)	Non-disabled household n (%)	Total n (%)	Disabled household n (%)	Non-disabled household n (%) (reference group)	Total n (%)	Disabled household n (%)	Non-disabled household n (%)	Total n (%)
***During the Ebola outbreak, did your community change how they acted towards anyone/any groups of people?***									
Yes (who and how described below)	206 (78%)**	96 (33%)**	302 (55%)	338 (51%)**	150 (23%)	488 (37%)	23 (55%)**	6 (9%)	29 (27%)
No	59 (22%) ††	192 (67%) ††	251 (45%)	321 (49%) ††	511 (77%)	832 (63%)	19 (45%) ††	58 (91%)	77 (73%)
***Which groups of people were treated differently***?									
Orphaned children whose parents died because of Ebola	149 (73%)**	37 (38%)	186 (62%)	119 (35%)	86 (57%)	205 (42%)	3 (13%)	2 (33%)	5 (17%)
Relatives of people who died because of Ebola	24 (12%)**	15 (15%)**	39 (13%)	16 (5%)	4 (3%)	20 (4%)	1 (4%)	1 (17%)	2 (7%)
Survivors of Ebola	14 (7%)**	18 (19%)**	32 (11%)	18 (5%)	7 (5%)	25 (5%)	8 (35%)**	1 (17%)	9 (31%)
Relatives of survivors of Ebola	0 (0%)	21 (22%)**	21 (7%)	3 (1%)	4 (3%)	7 (1%)	0 (0%)	0 (0%)	0 (0%)
Health workers	5 (2%)	1 (1%)	6 (2%)	26 (8%)	19 (13%)	45 (9%)	2 (9%)	0 (0%)	2 (7%)
Persons with disabilities	7 (3%)*	0 (0%)	7 (2%)	89 (26%)**	1 (1%)	90 (18%)	3 (13%)*	0 (0%)	3 (10%)
Sick people	0 (0%)	0 (0%)	0 (0%)	21 (6%)	0 (0%)	21 (4%)	4 (17%)	0 (0%)	4 (14%)
***How were they treated differently***?									
They were not allowed to return home	94 (46%)**	28 (29%)	122 (41%)	97 (28%)	85 (58%)	182 (37%)	2 (9%)	1 (25%)	3 (11%)
They were isolated in the community	72 (35%)**	24 (25%)**	96 (32%)	90 (26%)**	13 (9%)	103 (21%)	7 (30%)**	2 (50%)	9 (33%)
They were treated as an outsider	23 (11%)**	10 (10%)**	33 (11%)	61 (18%)**	3 (2%)	32 (7%)	12 (52%)**	0 (0%)	12 (44%)
They were rejected and shunned by others or treated as inferior	4 (2%)	4 (4%)	8 (3%)	29 (9%)**	3 (2%)	32 (7%)	1 (4%)	0 (0%)	1 (4%)
They were not treated fairly	6 (3%)	22 (23%)	28 (9%)	28 (8%)	22 (15%)	50 (10%)	0 (0%)	1 (25%)	1 (4%)

** *p*<0.0005 *p<0.005 significant increase in odds †† *p*<0.0005 †*p*<0.005 decrease in odds of outcome compared to reference group of Non-disabled households in no Ebola cases area, in multi-level mixed effects logistic regression adjusting for age, sex, education, and wealth quintile and clustering by village

Respondents from disabled households in all areas were significantly more likely to report people with disabilities were treated differently during the outbreak. Respondents in Ebola-affected areas (both non-disabled and disabled) were significantly more likely to report that relatives of people who died because of Ebola were treated differently (Table 6). However, only non-disabled household in Ebola-affected areas were significantly more likely to report that relatives of Ebola survivors were treated differently.

People with disabilities were asked whether they themselves were treated differently during the Ebola outbreak. Notably, only people with disabilities in Ebola-affected areas felt that they were treated differently (Table 7, fully adjusted models). While this may reflect the overwhelming significance of the Ebola outbreak, this also may also be due to wider discriminatory social norms towards disability that existed before the outbreak. Some people with disabilities interviewed reported not feeling included in their communities in general:

*“Once you are a disabled person, family and community members feel that you are a burden on them, they can say all the worse things to you because you cannot afford to gain that respect given to others who provide for the family” (Female respondent with disabilities, Greenvile, Sinoe)*

**Table 7:** People with disabilities perceptions of attitudes during Ebola

	A few, or many, cases of Ebola (*n*=560)			No Ebola cases (*n*=1343)			Don’t know whether there were any Ebola cases (*n*=107)		
	Disabled household n (%)	Non-disabled household n (%)	Total n (%)	Disabled household n (%) (reference group)	Non-disabled household n (%)	Total n (%)	Disabled household n (%)	Non-disabled household n (%)	Total n (%)
**[For disabled people only]** ***During the Ebola outbreak, did people change how they acted towards you?***									
Yes	76 (84%)**			108 (62%)			5 (56%)		
No	10 (11%)			65 (37%)			2 (22%)		
**[For disabled people only] - How did the way they act change?**									
I was not allowed to return home	13 (17%)*			2 (2%)			0 (0%)		
I was isolated in the community	24 (32%)			51 (47%)			1 (20%)		
I was treated as an outsider	15 (20%)*			11 (10%)			4 (80%)		
I was rejected and shunned by others or treated as inferior	8 (11%)			17 (16%)			0 (0%)		
I was not treated fairly	12 (16%)			18 (17%)			0 (0%)		

** *p*<0.0005 *p<0.005 significant increase in odds †† *p*<0.0005 †*p*<0.005 decrease in odds of outcome compared to reference group of disabled households in no Ebola cases area, in multi-level mixed effects logistic regression adjusting for age, sex, education, and wealth quintile and clustering by village

Disabled respondents in Ebola affected areas were significantly more likely to report changes in how people acted towards them during the Ebola outbreak compared with disabled respondents in non-affected areas (Table 7, fully adjusted models): they were not allowed to return home or were treated as outsiders.

Disabled respondents in all areas (Ebola affected, unaffected, unknown) were significantly more likely to report that life had become worse and that they were less happy than non-disabled respondents in unaffected areas (Table 8, fully adjusted models). Disabled respondents in Ebola-affected, and unaffected areas were also significantly more likely to report that, compared to the time before Ebola, they had less money than non-disabled respondents in unaffected areas (Table 8).

**Table 8:** Wellbeing compared to before the Ebola outbreak

	A few, or many, cases of Ebola (*n*=560)			No Ebola cases (*n*=1343)			Don’t know whether there were any Ebola cases (*n*=107)		
	Disabled household n (%)	Non-disabled household n (%)	Total n (%)	Disabled household n (%)	Non-disabled household n (%) (reference group)	Total n (%)	Disabled household n (%)	Non-disabled household n (%)	Total n (%)
***Compared to your life before the Ebola is your life…***									
Much better	10 (4%) ††	14 (5%) ††	24 (4%)	33 (5%) ††	114 (17%)	147 (11%)	2 (5%)	11 (17%)	13 (12%)
A bit better	102 (38%)	60 (21%)	162 (29%)	300 (45%)**	239 (36%)	539 (40%)	17 (40%)	16 (25%)	33 (31%)
No change	97 (36%)	194 (67%)	291 (52%)	171 (25%) ††	274 (41%)	445 (33%)	15 (35%)	33 (52%)	48 (45%)
A bit worse	48 (18%)**	21 (7%)	69 (12%)	101 (15%)**	37 (6%)	138 (10%)	7 (16%)*	2 (3%)	9 (8%)
Much worse	9 (3%)	2 (1%)	11 (2%)	67 (10%)**	3 (0.5%)	70 (5%)	2 (5%)*	2 (3%)	4 (4%)
Mean^2^ (SD)	2.79 (0.90)	2.78 (0.67)	2.79 (0.79)	2.81 (1.08)	2.36 (0.84)	2.58 (0.99)	2.76 (0.95)	2.5 (0.93)	2.61 (0.94)
***Compared to your life before the Ebola outbreak do you have…***									
Much more money	3 (1%)	2 (1%)	5 (1%)	10 (1%) †	24 (4%)	34 (3%)	0 (0%)	3 (5%)	3 (3%)
A bit more money	80 (30%)	48 (16%)	128 (23%)	225 (24%)	207 (31%)	432 (32%)	13 (30%)	16 (25%)	29 (27%)
No change	104 (39%) ††	211 (72%)	315 (56%)	210 (31%) ††	362 (54%)	572 (43%)	19 (44%)	37 (58%)	56 (52%)
A bit less money	49 (18%)**	22 (8%)	71 (12%)	105 (16%)**	46 (7%)	151 (11%)	7 (16%)	4 (6%)	11 (10%)
Much less money	29 (11%) ††	10 (3%)	39 (7%)	121 (18%) ††	27 (4%)	148 (11%)	4 (9%)	4 (6%)	8 (7%)
Mean^2^ (SD)	3.08 (0.98)	2.97 (0.63)	3.02 (0.82)	3.15 (1.12)	2.77 (0.80)	2.96 (0.99)	3.05 (0.92)	2.84 (0.86)	2.93 (0.89)
***Compared to your life before the Ebola outbreak are you…***									
Much more happy	16 (6%) ††	45 (15%) †	61 (11%)	98 (15%) ††	285 (43%)	383 (29%)	2 (5%) ††	24 (38%)	26 (25%)
A bit more happy	106 (40%)	71 (24%) ††	177 (32%)	259 (39%)	296 (45%)	555 (42%)	17 (40%)	24 (38%)	41 (39%)
No change	95 (36%)**	152 (52%)**	247 (44%)	138 (21%)**	52 (8%)	190 (14%)	16 (37%)**	10 (16%)	26 (25%)
A bit more unhappy	33 (12%)**	21 (7%)	54 (10%)	85 (13%)**	27 (4%)	112 (8%)	5 (12%)	5 (8%)	10 (9%)
Much more unhappy	16 (6%)**	3 (1%)	19 (3%)	91 (14%)**	5 (1%)	96 (7%)	3 (7%)*	0 (0%)	3 (3%)
Mean^2^ (SD)	2.73 (0.97)	2.54 (0.87)	2.63 (0.92)	2.72 (1.25)	1.75 (0.82)	2.24 (1.16)	2.77 (0.97)	1.94 (0.93)	2.27 (1.03)

** *p*<0.0005 *p<0.005 significant increase in odds †† *p*<0.0005 †*p*<0.005 decrease in odds of outcome compared to reference group of Non-disabled households in no Ebola cases area, in multi-level mixed effects logistic regression adjusting for age, sex, education, and wealth quintile and clustering by village

^1^ Responses chosen by >10% of respondents in at least one sub-population (column) shown only; column percentages

^2^ 'Much better'=1, 'A bit better'=2, 'No change'=3, 'A bit worse'=4, 'Much worse'=5

## Discussion

Our findings show that despite limited efforts, people with disabilities were largely left out of Ebola responses due to lack of targeted or deliberate inclusion efforts. Partly this was because of pre-existing inequalities long experienced by disabled Liberians. This also reflected a lack of understanding from professionals about disability inclusion, compounded by a lack of disability-specific funding, training or even awareness across all levels of the health system and wider society. The resulting exclusion has limited the recovery of people with disabilities from the health, social and economic consequences of the epidemic, and has implications for future responses to disease outbreaks, as well as those newly disabled by EVD [14].

These findings are in line with other studies exploring general healthcare access for persons with disabilities (e.g. [7, 22]). Both studies also note the lack of consensus around what healthcare access looks like for people with disabilities. Assessing the impact of Ebola on people with disabilities, a number of issues stand out. First is how pre-existing inequalities impacted households of people with disabilities in terms of responses to public health advice. For example, while many disabled households followed public health advice and reduced the number of visitors, they did not particularly reduce eating with others, or going out. Many disabled households remain reliant on begging or on charitable donations of food and goods, necessitating the need to go out into the community even though they knew they were at risk. In addition to direct health consequences, the necessary public health measures the government took in response to the pandemic (e.g. lockdowns, curfews, business closures, restrictions on movement) impacted everyone, but were particularly acute for people with disabilities, resulting in reduced social and economic support for disabled households, and lack of access to information. Others have also noted an increased risk of loneliness and even intra-household violence [18]. Moreover, our interviews and FDGs found that people with disabilities were largely excluded from activities intended to support the regeneration of the social and economy networks after the EVD outbreak further perpetuating these inequalities.

Second, members of disabled households were less likely to go to clinics or hospitals for treatment if they did become sick. This may have been due to cost, with hospital care perceived as too expensive. Many, especially women with disabilities, sought help from traditional healers, or attempted to self-treat themselves and their families. There were also significant fears about entering Ebola treatment units (ETU) during the outbreak, as well as fears about stigma and exclusion [27]. Moreover, if basic services like maternal and child health  and malaria interventions were so significantly reduced, it can be assumed that the minimal services and supports previously available for people with disabilities (including those run by international NGOs who left the country) were significantly reduced or eliminated in face of Ebola.

Third, the lack of preparedness of health services for disability inclusion at all levels – such as lack of Sign Language interpreters, inaccessible building and accessible transport options – is striking. Despite some advocacy activities to raise awareness by organisations of people with disabilities and other stakeholders (c.f.: [13]), it is evident that healthcare workers were woefully unprepared and untrained on all aspects of disability inclusion, despite the Government of Liberia’s disability commitments, as evidenced by the National Commission on Disability Act 2005 and National Action Plan on Disabilities 2018. This lack of training meant treatment and access for people with disabilities was ad hoc, often an after-thought and largely reliant on individual public health officials or clinical practitioners.

Households of people with disabilities in both exposed and non-exposed areas reported primarily getting information from community leaders, rather than government and/or local authority representatives, and in area exposed to EVD, households of people with disabilities were less likely to listen to health workers than other households. This may indicate a lack of trust of the healthcare system (or government more broadly), but also calls into question whether the information received is accurate, timely or trusted. It could also be that information was not given in formats accessible to many.

Another important finding is that many people with disabilities were unable to work, especially when this involved begging, and there is very little in the way of social protection services to fill this gap. This left them at reliant upon limited charitable resources, which is against the human rights commitments made by the Government of Liberia.

This brings us to the issue of trust. As related studies show, in Liberia, as elsewhere, people with disabilities have often been let down by the state, with promises unfulfilled and action plans postponed [17]. There is a need for the Government of Liberia and service providers to agree on who is responsible for disability issues. Currently the Ministry of Gender, Children and Social Protection holds overall responsibility, which led to lack of focus, mainstreaming or staff training. Moreover, there is a lack of coordination of organisations representing people with disabilities, and limited inclusion at local, national and international levels. This diminishes the capacity to advocate effectively. This is particularly challenging in an environment where these organisations represent a range of different impairment types, leading to conflicting priorities amongst organisations with limited resources.

Melissa Leach has previously talked about this lack of trust, and how states might go about addressing it. The first is through a multidimensional ‘inclusive security’ approach, which recognises and addresses political disenfranchisement and claims for justice, as well as (re)building institutions. Key to this is how these institutions build trust, equality and security [20]. In more recent work, she and colleagues note that trust is “...both a measure of state-citizen relations and an enabler of effective response to diseases and development more general” ([21]: 8). Trust is mediated by a range of factors, including history, conflict, the state, and levels of equality within societies. Rebuilding trust requires not only improved services, but improved dialogue (as happened during the Ebola response) ([21]: 8)

### Long term impacts and the COVID-19 pandemic

A post-Ebola review noted the “profound and multifaceted” negative impact of EVD on the Liberian health system with an associated breakdown of trust in the health system, and substantial reductions in healthcare utilisation [11]. This breakdown included substantial reductions in maternal delivery care in Ebola-affected areas, childhood malaria admissions and vaccination coverage (ibid). Increased morbidity and mortality and reduced life expectancy were also reported. It can be speculated that if basic services like maternal and child healthcare and malaria treatments were so significantly reduced whatever limited services there were for people with disabilities may have also been significantly reduced or even eliminated during the Ebola epidemic. Additionally, the Ebola epidemic has increased the in numbers of children and adults with potentially disabling conditions, stretching already limited resources even tighter. A similar pattern is likely to be observed with the current COVID-19 response [2]

Our findings on Ebola, disability and inequality have further implications in light of the current COVID-19 pandemic. These are consistent of those with Kelley et al. [15] who argue that the COVID-19 pandemic can build on lessons learned from global Cholera and Ebola outbreaks in West Africa. Furthermore, responses to the long-term disabling consequences of EVD [11, 14] also have relevance to COVID-19. Many survivors will require long-term health and rehabilitation services, and psychological support - services which are largely unavailable in Liberia. This will put additional pressure on an already fragile health system.

The lack of coordination between Government ministries highlighted above has implications for the inclusivity of the ongoing COVID-19 response in Liberia. People with disabilities in Liberia may be at increased risk COVID-19 as our research found that they are often unable to remain at home as per public health advice as they have little alternative but to beg or get charitable donations from members of the community. People with disabilities are at risk of falling between the gaps and missing out on vital healthcare and other services - further exacerbating entrenched inequities, including in the current COVID-19 response activities. Our findings also indicate a more general lack of trust in the healthcare system (particularly how information is shared). This has important implications for how public health information related to any subsequent epidemic, including COVID-19 is being passed on to communities in Liberia.

For people with disabilities, this rebuilding of trust needs to include provision of adequate social protection, recognition of rights and justice, as well as equality of access to goods and services - all of which were in need before the COVID-19 pandemic and have become markedly worse as the pandemic continues [29].

## Conclusion

The results presented here highlight how people with disabilities in Liberia experienced the Ebola outbreak. They demonstrate how pre-existing inequalities and exclusions were exacerbated, and new ones created by the outbreak, largely by the public health response itself. However, while the response to the EVD epidemic did not address issues of social justice for people with disabilities living in low-income countries, as highlighted above there are some lessons that can be learned for national and international responses to the current COVID-19 pandemic (and other infectious diseases), as well as for longer term recovery and rehabilitation to avoid exacerbating and further entrenching existing inequalities.

Key findings show the need to understand and mitigate direct and indirect impacts of the epidemic on people with disabilities and the necessity to significantly increase the capacity of healthcare workers and other social services to respond to their needs and rights. Reflections by people with disabilities themselves clearly show how their knowledge about, preparation for and responses to the EVD epidemic were often markedly different because of lack of resources, and lack of inclusion in many mainstream public health, medical, social and economic initiatives. This exclusion largely reflects persistent discrimination and marginalisation of people with disabilities in Liberia, which not only impacted this population during the Ebola epidemic but has implications for the ongoing COVID-19 pandemic.

Understanding wider issues around equity and social justice for marginalised and excluded groups can be seen as a key “technology” of epidemic control – one that also addresses racial, gendered, and political struggles ([24]: 467). In this paper we have explored the responses and experiences of people with disabilities in Liberia during the Ebola outbreak and what lessons can be learned on disability inclusion. This includes early responses and interventions, and prioritisation for treatment and resources. Exclusion of people with disabilities from these is a breach of human rights. While the current COVID-19 pandemic is markedly different from the Ebola outbreak, there are lessons to be learned from Ebola around timely and equitable inclusion at all phases of the response. There is urgent need to rethink how to deliver and adequately fund equitable and inclusive immediate and long-term services and support. Mechanisms for accountability need to be strengthened, which in turn may help build trust in government. Finally, people with disabilities need to be included in all aspects of the response. Now is the time for such measures to ensure people with disabilities are not further marginalised in global responses to pandemics.

### Limitations

The research had two main limitations. First, participants with disabilities were identified through a list provided by the national umbrella organisation of people with disabilities (National Union of Organisations of the Disabled - NUOD). People with disabilities who are in contact with an established organisation may have higher levels of wellbeing in some domains (e.g., educational attainment, political engagement, and community relations), compared to those not in regular contact. Gaps in relative inequalities may therefore be larger for people with disabilities who are not members of the organisation. Second, as the survey is a cross-sectional study, the results provide a detailed ‘snapshot’ of multidimensional poverty between disabled and non-disabled Liberians, but we are unable to identify whether and how this might change over time.

Respondents were not asked whether they were survivors of EVD. It was decided that due to the continuing stigma surrounding the disease this would lead to substantial underreporting.

## Supplementary Information

**Additional file 1.**

## Data Availability

Data sets used in this research can be made available on request
