# Supplementary material for: The Ebola crisis and people with disabilities’ access to healthcare and government services in Liberia
Source: Int J Equity Health. 2021 Nov 24;20:247. doi: 10.1186/s12939-021-01580-6 (PMC8611399; doi:10.1186/s12939-021-01580-6)
Supplement: Supplementary file 1 — Additional file 1. [file 12939_2021_1580_MOESM1_ESM.pdf]

RESPONDENT DETAILS

Before we begin, we would first like to ask you to answer a few general questions about you and your household.

**A1**	**Gender *[circle one option]***				
	Male	1	Female	2	
**A2**	**Age at last birthday (years)**				
**A2_1**	**Were you born in [location]? *[circle one option]***				
	Yes (go to A3)	1	No (go to A2_2)	2	
**A2_2**	**If no, WHERE were you born?**				
	**If no, WHEN did you move in [location] (year)?**				
**A2_3**	**If no, WHY did you move to [location]? *[circle as many as apply]***				
	Marriage	1	Divorce/separation	5	
	Work	2	Other (please specify)	6	
	Displaced by war	3	Don't know	88	
	Ebola	4	Refused answer	99	
*****QUESTIONS A3 to A3_4 <u>ONLY</u> ASK HEAD OF HOUSEHOLD. OTHERWISE GO TO A4_1*****					
**A3**	**Total number in household**	**Adults**		**Children** (under 17)	
**A3_1**	**How many rooms are in your household?**				
**A3_2**	**Does the household own the dwelling? *[circle one option]***				
	Owns the dwelling	1	Other (please specify)	5	
	Rents the dwelling	2	Don't know	88	
	Uses without paying rent	3	Refused to answer	99	
	Nomadic or temporary	4			
**A3_3**	**Does the household own any land? *[circle one option]***				
	Yes (go to A3_4)	1	No/don't know (go to A4_1)	2	
**A3_4**	**How many lots does the household own? *[write in number of lots]*** (4 lots = 1 acre)	__________	Don't know	-88	
**A3_5**	**What is the land used for? *[don't read the options, circle as many as apply]***				
	Household lives on the land	1	Not used for anything	6	
	Farmed by the household	2	Used for business purposes	7	
	Rented out for farming	3	Other (please specify)	8	
	Renting out for housing	4	Don't know	88	
	Rented for business purposes	5	Refused to answer	99	
**A4_1**	**Relationship of respondent to the head of household *[circle one option]***				
	Is head of household	1	Niece/nephew	6	
	Spouse	2	Grandparent	7	
	Son/daughter	3	Grandchild	8	
	Brother/sister	4	Non-relative/family member	9	
	Parent	5	Other (please specify)	10	

**A4_2**	**Marital status *[circle one option]***			
	Single	1	Separated	6
	Married monogamous	2	Divorced	7
	Married polygamous	3	Widowed / Widower	8
	Living together (not married)	4	Other (please specify)	9
	In a relationship (not living with partner)	5		
**A4_3**	**Religion [*circle one option]***			
	Christian	1	Other (please specify)	4
	Muslim	2		
	Traditional beliefs	3	No religion	5
**A4_4**	**What is your ethnic affiliation? *[circle one option]***			
	Bassa	1	Krahn	11
	Belle	2	Kru	12
	Dey	3	Lorma	13
	Gbandi	4	Mandingo	14
	Gbi	5	Mano	15
	Gio	6	Mende	16
	Gola	7	Sapo	17
	Grebo	8	Vai	18
	Kpelle	9	Congo Liberian/Americo-Liberian	19
	Kissi	10	Naturalised Liberian	20

**A5**	**Thinking about your own life and personal circumstances, how satisfied are you with your life as a whole? *[read out the options, circle one option]***				
	Not at all satisfied	a bit unsatisfied	not satisfied or unsatisfied	a bit satisfied	completely satisfied
	1	2	3	4	5

**A6**	**What do you think are the three most important things to live a life with dignity? *[read options, and write 1, 2, 3 next to the chosen ones, where 1 = most important]***			
	To have a job		To live in a country with no war	
	To have good health		To live in a country without violence	
	To have a good living standard		To live in a country without corruption	
	To get an education (myself)		To have a happy family life	
	To get a good education for my children		To live in freedom	
	To be able to participate in public life		To live free of fear	
	To live a life free of hunger		To live free of want	
	To live independently/ have my own home		To live in a country with an effective/good justice system	
	To be faithful to my religion		Other (please specify)	

HOUSEHOLD DETAILS

Next we would like to ask you to answer some questions on your standard of living.

	ONLY ASK B1_1 AND B1_2 TO HEAD OF HOUSEHOLD, OTHERWISE GO TO B1_3			
**B1_1**	**Does your household have any of the following? *[read list – circle all that apply]***			
	Electric iron	1	Mosquito net/bed net	12
	Charcoal iron	2	Mattress	13
	Refrigerator	3	Bed	14
	Deep freezer	4	Watch or clock	15
	Television	5	Sewing machine	16
	VCR/DVD	6	Modern Stove	17
	Radio	7	Canoe/boat	18
	Mobile/cell phone	8	Bicycle	19
	Computer	9	Motorcycle	20
	Generator	10	Car or truck	21
	Fan	11		
**B1_2**	**What is the main source of drinking water for your household? *[circle one option]***			
	Piped into residence/compound (go to B1_5)	1	Rainwater	8
	Public tap/standpipe	2	River/stream	9
	Hand pump in residence/ compound (go to B1_5)	3	Tanker/truck	10
	Well in residence/compound (go to B1_5)	4	Pond/lake	11
	Bought bottled water	5	Still water	12
	Open well	6	Other (please specify)	13
	Spring	7		
**B1_3**	**How long does it take you to walk to the nearest supply of drinking water? *[write in number]***			________ minutes

**B1_4**	**How safe is it for women, children, persons with disabilities and elders to reach to the nearest supply of drinking water? *[read out the options, circle one option]***				
	Very safe	a bit safe	neither safe nor unsafe	a bit unsafe	very unsafe
	1	2	3	4	5

	ONLY ASK B1_5 to B1_7 to HEAD OF HOUSEHOLD. OTHERWISE GO TO B1_8			
**B1_5**	**What kind of toilet facility does your household have? *[circle one option]***			
	Private flush inside	1	Traditional pit	4
	Private flush outside	2	Open defecation near to the house (e.g. beaches or bushes)	5
	Shared flush (communal)	3	Other (please specify)	6

**B1_6**	**What is the <u>main</u> cooking fuel *[circle one option]***			
	Firewood	1	Crop residue/sawdust	6
	Charcoal	2	Animal waste	7
	Kerosene/oil	3	Other (please specify)	9
	Gas	4	Don't know	88
	Electricity	5	Refused answer	99
**B1_7**	**What is the <u>main</u> source of light the household? *[circle one option]***			
	Firewood	1	Solar powered lamp	6
	Kerosene/oil lamps	2	Candles	7
	Gas	3	Other (please specify)	8
	Electricity	4	Don't know	88
	Battery powered lamp / light	5	Refused answer	99
**B1_8**	**How many meals per day do you usually eat? *[write number]***			_____
**B1_9**	**How many meals did you eat yesterday? *[write number]***			_____
**B1_10**	**How often do you get enough to eat? *[read out the options, circle one option]***			
	Always enough	1	Often not enough	3
	Often enough	2	Never enough	4
**B1_11**	**In the last month, did any member of your household have nothing to eat for one or more days?**			
	Yes (go to B1_12)	1	No/don't know (go to B1_13)	2
**B1_12**	**How many days did the member of your household have nothing to eat? *[write number]***			____________
			Don't know how many days	-88

ONLY ASK B1_13 to B1_17 TO HEAD OF HOUSEHOLD. OTHERWISE GO TO B2_1						
**What is the condition of the dwelling... *[read out the options, circle one option]***		**Very good**	**a bit good**	**a bit poor**	**very poor**	**do not have**
**B1_13**	Walls?	1	2	3	4	8
**B1_14**	Floors?	1	2	3	4	8
**B1_15**	Roof?	1	2	3	4	8
**B1_16**	Windows?	1	2	3	4	8
**B1_17**	Doors	1	2	3	4	8

**B2_1**	**Not at all satisfied**	**a bit unsatisfied**	**neither satisfied nor unsatisfied**	**a bit satisfied**	**completely satisfied**
How satisfied are you with your own standard of living? ***[read out the options, circle one option]***	1	2	3	4	5

B2_2	Living with a lot of difficulty	Living with a bit of difficulty	managing	living a bit well	living very well
How well do you live based on your current household income? ***[read out the options, circle one option]***	1	2	3	4	5

B2_3	**In the last year has your household living standard… *[read out the options, circle one option]***			
	Got better	1	Don't know	88
	Stayed the same	2	Refused question	99
	Got worse	3		

B2_4	Poorer	Fairly poor	in the middle	Fairly rich	Richer
How would you compare your standard of living to other households in your community? ***[read out the options, circle one option]***	1	2	3	4	5

HEALTH AND HEALTHCARE SERVICES

Next we would like to ask you about your health and access to health services

C1	Not at all satisfied	a bit unsatisfied	neither satisfied or unsatisfied	a bit satisfied	completely satisfied
How satisfied are you with your health overall? ***[read out the options, circle one option]***	1	2	3	4	5

[For C2_1 to C2_7 read out the options, circle one option. If the response is some difficulty, a lot of difficulty or cannot do at all, write the age the respondent started to have difficulty. From birth, write 0]		No difficulties	With some difficulty	With a lot of difficulty	Cannot do at all	Since when (age)
**C2_1**	Do you have difficulty seeing, even if wearing glasses?	1	2	3	4	____
**C2_2**	Do you have difficulty hearing, even if using a hearing aid?	1	2	3	4	____
**C2_3**	Do you have difficulty walking or climbing steps?	1	2	3	4	____
**C2_4**	Do you have difficulty raising a 2 litre bottle of water or soda from waist to eye level?	1	2	3	4	____
**C2_5**	Do you have difficulty remembering or concentrating?	1	2	3	4	____
**C2_6**	Do you have difficulty with self-care such as washing all over or dressing?	1	2	3	4	____
**C2_7**	Do you have difficulty understanding or being understood?	1	2	3	4	____

**C2_8**	**How often do you feel worried, nervous or anxious? *[read out the options, circle one option]***			
	Every day	1	Never	5
	Once a week	2	Don’t know	88
	Once a month	3	Did not answer	99
	A few times a year	4		
**C2_9**	**Do you have fits (jerking body movements) or does your body go rigid? *[read out the options, circle one option]***			
	Never	1	Often	3
	Occasionally	2	All the time	4
**C3_1**	**Do you need any devices or support to get around? *[circle one option]***			
	Yes (go to C3_2)	1	No (go to C4_1)	2
**C3_2**	**What devices or support do you need to get around? *[DO NOT read out the options, circle as many as apply]***			
	Walking stick	1	Hearing aid	7
	Crutches	2	Glasses	8
	Wheelchair	3	White cane (visual impairment)	9
	Tricycle	4	Communication aids	10
	Artificial limb*	5	Other (please specify)	11
	Someone’s assistance	6		
**C3_3**	**Who helps you move around/mobilise on a regular basis? *[DO NOT read out the options, circle as many as apply]***			
	No one	1	Other adult male	6
	Male adult household member	2	Other adult female	7
	Female adult household member	3	Other male child	8
	Male child household member	4	Other female child	9
	Female child household member	5		
**C3_4**	**How often do you have access to these devices or support? *[circle one option]***			
	All the time (go to C4_1)	1	Occasionally (go to C3_5)	3
	Most of the time (go to C3_5)	2	Never (go to C3_5)	4
**C3_5**	**Why don’t you have the devices you need to get around? *[DO NOT read out the options, circle as many as apply]***			
	They cost too much	1	They are not available	3
	I don’t know where to find them	2	Other (please specify)	4

**C4_1**	**Not at all satisfied**	**a bit unsatisfied**	**neither satisfied or unsatisfied**	**a bit satisfied**	**completely satisfied**
How satisfied are you with your access to health services? ***[read out the options, circle one option]***	1	2	3	4	5

**C4_2**	**How often can you get the healthcare you need?** ***[read the options, circle one option]***			
	All the time	1	Occasionally/sometimes	3
	Most of the time	2	Never	4
**C4_3**	**Where would you go in the case of a health problem or accident?** ***[DO NOT read out the options, circle as many as apply]***			
	Private clinic or hospital	1	Private doctor/dentist	8
	Government clinic/health centre	2	Health NGO	9
	Government hospital	3	Neighbour	10
	Pharmacy	4	Other (please specify) ______________________	11
	Traditional medicine/herbalist	5	Don't know	88
	Mobile clinic/drug peddler	6	Refused answer	99
	Religious leader	7		
**C4_4**	**Why don't you have access to healthcare?** ***[skip if C4_2 = all the time. DO NOT read out the options, circle as many as apply]***			
	No need	1	Services are not available/lack of facilities	7
	It costs too much	2	Lack of adequate medication/drugs/supplies	8
	I don't know where it is	3	The staff treat me badly/unfriendly staff	9
	Don't know the normal opening hours	4	Long waiting times/takes too long	10
	The health facility is not accessible to me (no ramps, sign language etc.)	5	Other (please specify)	11
	It is too far away/long distance to health facility	6		
**C4_5**	**Do you have problems when you visit the health facility in your community?** ***[skip if C4_2 = never. DO NOT read out the options, circle as many as apply]***			
	No problems/satisfied	1	Treatment unsuccessful	10
	Facility is not clean	2	Long distance to health facility	11
	Long waiting times to see a health service provider	3	No drugs/medications available (drug stock out, lack of medicine)	12
	I went to the health facility and they denied me attention	4	The staff treat me badly/unfriendly staff	13
	Services not in the local language	5	The health facility is not accessible to me (no ramps, sign language etc.)	14
	No ambulance	6	No female health professionals	15
	Lack of privacy in examination room	7	The facility does not offer the correct treatment	16
	No medical doctor/ trained professionals	8	Other (please specify)	17
	I do not have money/too expensive (or high cost of medicine/fees)	9		

**C4_6**	**How would you <u>usually</u> get to your health facility? *[circle* ONE *option only*]**			
	Walk	1	Motorbike / Pempem	6
	Government owned bus	2	Tricycle / Keke	
	Government ambulance	3	Car taxi	7
	Private car	4	Other (please specify):________________	8
	Bicycle	5	Don't know	88
**C4_7**	**Using the above transport, how long does it take to reach your nearest health facility? *[write the number]***		_____________ (minutes)	
	Don't know	-88	Refused answer	-99
**C4_8**	**Does the health facility have a private examination room that you can use? *[circle one option]***			
	Yes	1	Don't know	88
	No	2	Refused answer	99
**C4_9**	**Does the facility charge user fees for health services? *[circle one option]***			
	Yes (go to C4_10)	1	Don't know (go to C4_11)	88
	No (go to C4_11)	2	Refused answer (go to C4_11)	99
**C4_10**	**Which services does the facility charge for? *[read out the options, circle as many as apply]***			
	Registration	1	Laboratory Test	4
	Consultation	2	Other (please specify)	5
	Treatment/Drugs	3		
**C4_11**	**Did you pay for your last health consultation? *[circle one option]***			
	Yes	1	Don't know	88
	No	2	Refused answer	99
**C4_12**	**How much do you <u>usually</u> spend on your health per month? *[write the number]***		___________________ (Liberian $)	
	Don't know	-88	Refused answer	-99

**C4_13**	How satisfied are you with the health care you receive? ***[read out the options, circle one option]***	**Not at all satisfied**	**a bit unsatisfied**	**neither satisfied or unsatisfied**	**a bit satisfied**	**completely satisfied**
		1	2	3	4	5

**C5_1**	**Were there any Ebola cases in your community? *[circle one option]***			
	Yes, a few cases	1	No Ebola cases	3
	Yes, many cases	2	Don't know	88
**C5_2**	**Did the Ebola quarantines affect your household/community? *[circle one option]***			
	Yes, my household was quarantined	1	No	4
	Yes, households in my community were quarantined (not mine)	2	Don't know	88
	Yes, the entire community was quarantined	3	Refused answer	99

C5_3	**How did Ebola affect you and your community?** ***[DO NOT read out the options, circle as many as apply]***			
	decreased social life in the community	1	people didn't have money for food	10
	stopped communal eating or eating in the same place	2	restricted holding of community meetings	11
	stopped welcoming visitors and strangers in the home	3	affected traditional culture and practices	12
	restricted movement of persons in and out of the community	4	changed the way people took care of the sick in the family and community	13
	many orphaned children	5	Loss of job/livelihood	14
	community members did not seek health treatment at health facilities for fear of being isolated or rejected	6	negative treatment and stigma of Ebola affected people (e.g. orphans, survivors and relatives)	15
	close down of health facilities	7	Don't know	88
	close down of schools	8	Refused answer	99
	people didn't have enough money to send children back to school	9	Other (please specify)	16
C5_4	**Who did you and your community listen to when making decisions about how to stop the spread of Ebola?** ***[DO NOT read out the options, circle as many as apply]***			
	community leaders	1	Family members	7
	religious leaders	2	Friends/neighbours	8
	traditional leaders	3	Government/local authority representatives	9
	disabled people's organizations (DPOs)	4	Other (please specify) ___________________	10
	Teachers/school administrators	5	Don't know	88
	health workers	6		
C5_5	**What were the main problems you and your community faced during the Ebola outbreak?** ***[DO NOT read out the options, circle as many as apply]***			
	lack of information on the Ebola outbreak	1	worse sanitation	10
	lack of information on types of health services available in health facilities	2	lack of access to medical care/essential drugs/vaccines	11
	Closure of health facilities	3	Children out of school	12
	lack of access to Ebola Treatment Units (ETU)	4	Loss of job/livelihood	13
	lack of food	5	Unfriendly attitude of health workers	14
	lack of safe drinking water	6	not able to bury loved ones in the usual way	15
	lack of access to water, soap and chlorine	7	People afraid of going to health facilities fearing exposure to Ebola	16
	Loss of freedom during quarantine	8	Negative perception of quarantine as shameful	17
	lack of information on accessible services for vulnerable groups (e.g. persons with HIV/AIDS and/or TB or persons with disabilities)	9	Other (please specify)	18

**C5_6**	**In your opinion, who suffered the <u>most</u> during the Ebola outbreak? *[read out ALL the options, circle ONE option]***				
	orphaned children whose parents died because of Ebola	1	Community health volunteers	9	
	relatives of people who died because of Ebola	2	Persons with disabilities	10	
	survivors of Ebola	3	Persons with HIV/AIDS or TB	11	
	relatives of survivors of Ebola	4	Older people	12	
	Health workers	5	Religious leaders	13	
	Ambulance teams	6	Pregnant Women	14	
	Traditional healers	7	Other (please specify)	15	
	Burial teams	8	Don't know	88	
**C5_7**	**During the Ebola outbreak, did your access to health services *[read out the options, circle one option]***				
	Get better	1	Get worse	3	
	Stay the same	2	Don't know	88	
**C5_8**	**How did you get treatment during the Ebola outbreak?**				
	Did not need treatment	1	Other (please specify)	5	
	Health facilities	2			
	Traditional healers	3	Don't know	88	
	Self-treatment/treatment from household members	4	Refused answer	99	
**C5_9**	**During the Ebola outbreak, did your community change how they acted towards anyone/any groups of people?**				
	Yes (go to C5_10)	1	No/don't know (go to C6_1)	2	
**C5_10**	**Which groups/people were treated differently? *[read out ALL the options, circle as many as apply]***				
	orphaned children whose parents died because of Ebola	1	Community health volunteers	8	
	relatives of people who died because of Ebola	2	Persons with disabilities	9	
	survivors of Ebola	3	Persons with HIV/AIDS or TB	10	
	relatives of survivors of Ebola	4	Older people	11	
	Health workers	5	Religious leaders	12	
	Ambulance teams	6	Other (please specify)	13	
	Burial teams	7	Don't know	88	
**C5_11**	**How were they treated differently? *[DO NOT read the options, circle as many as apply]***				
	they were not allowed to return home	1	they were refused transportation in "collective" car taxis and "pen-pen" motorbikes	6	
	they were isolated in the community	2	they were stopped from returning to their regular jobs	7	
	they were treated as an outsider	3	the issue of being a survivor became a taboo	8	
	they were rejected and shunned by others or treated as inferior	4	Other (please specify)	9	
	they were not treated fairly	5	Don't know	88	

[only ask C5_12 and C5_13 if the respondent is a person with disabilities, otherwise go to C6_1]					
**C5_12**	**During the Ebola outbreak, did people change how they acted towards you?** ***[circle one option]***				
	Yes (go to C5_13)	1	Don't know (go to C6_1)	88	
	No (go to C6_1)	2	Refused question (go to C6_1)	99	
**C5_13**	**How did the way they act change?** ***[DO NOT read the options, circle as many as apply]***				
	I was not allowed to return home	1	I was refused transportation in "collective" car taxis and "pen-pen" motorbikes	6	
	I felt isolated in the community	2	I was stopped from returning to my regular jobs	7	
	I was treated as an outsider	3	the issue of being a survivor became a taboo	8	
	I felt rejected and shunned by others or treated as inferior	4	Other (please specify) ____________________	9	
	I was not treated fairly	5	Don't know	88	
**C6_1**	**Compared to your life before the Ebola is your life…** ***[read out the options, circle one option]***				
	Much better	A bit better	No change	A bit worse	Much worse
	1	2	3	4	5
**C6_2**	**Compared to your life before the Ebola outbreak do you have…** ***[read out the options, circle one option]***				
	Much more money	A bit more money	No change	A bit less money	Much less money
	1	2	3	4	5
**C6_3**	**Compared to your life before the Ebola outbreak are you…** ***[read out the options, circle one option]***				
	Much more happy	A bit more happy	No change	A bit more unhappy	Much more unhappy
	1	2	3	4	5

EDUCATION

Now I would like to ask you some questions about school and education

**D1**	**What is the highest level of education you have completed?** ***[circle one option]***			
	No formal education (go to D2_1)	1	Some College (go to D3_1)	6
	Some primary (go to D2_1)	2	Completed College (go to D3_1))	7
	Completed primary (go to D2_1)	3	Some university (go to D3_1)	8
	Some secondary (go to D2_1)	4	University (go to D3_1)	9
	Completed secondary (go to D3_1)	5	Other (specify) (go to D2_1)	10

[for D2_1 and D2_2 read the options, circle one option]		Well	With some difficulty	With a lot of difficulty	Cannot do at all
**D2_1**	Can you read and write?	1	2	3	4
**D2_2**	Can you do maths/sums?	1	2	3	4

**D3_1**	How satisfied are you with the education/ school in your community? ***[read the options, circle one option]***	**Not at all satisfied**	**a bit unsatisfied**	**neither satisfied or unsatisfied**	**a bit satisfied**	**completely Satisfied**	**Don't know**
		1	2	3	4	5	88
**D3_2**	How important do you think it is for a child to go to school? ***[read the options, circle one option]***	**Very unimportant**	**a bit unimportant**	**neither important or unimportant**	**a bit important**	**very important**	**Don't know**
		1	2	3	4	5	88
**D3_3**	How important is education for persons with disabilities? ***[read the options, circle one option]***	**Very unimportant**	**a bit unimportant**	**neither important or unimportant**	**a bit important**	**very important**	**Don't know**
		1	2	3	4	5	88

WORK AND EMPLOYMENT

Now I would like to ask you some questions about work and employment. By work and employment we mean a job where you get paid, your own business or income from farming.

**E1_1**	**What is your main source of income? *[DO NOT read out the options, circle one option]***			
	No income	1	Money sent home from abroad	7
	Formal paid employment	2	Money sent home from within Liberia	8
	Self-employed	3	Street begging	9
	Family support/donation	4	Government aid	10
	Other individual support (not from family/relatives)	5	Don't know	88
	Aid from international/civil society organisation	6	Other (please specify)	11
**E1_2**	**Do you receive any financial or welfare assistance?**			
	Yes (go to E1_3)	1	Don’t know (go to E1_4)	88
	No (go to E1_4)	2	Refused answer (go to E1_4)	99

**E1_3**	**What kind of assistance do you receive?** ***[DO NOT read out the options, circle as many as apply]***			
	National pension scheme	1	Social cash transfer	5
	Employment injury scheme	2	School take-home rations	6
	Survivor's pension	3	Other (please specify)	7
	Refund	4		

**E1_4**	**How much money do you make?** ***[fill in the number, circle week or month]***	__________ (Liberian $)<br><br>Per week / per month
	Don't know -88	Refused answer -99
	*****ONLY ASK E1_5 to HEAD OF HOUSEHOLD. OTHERWISE GO TO E1_6*****	
**E1_5**	**How many people in your household currently have a job where they get paid or have their own business or get income from farming?** ***[write the number]***	

**E1_6**	**Do you have a job where you get paid or your own business or income from farming?**			
	Yes. (go to E1_10)	1	No (go to E1_7)	2
**E1_7**	**Are you looking for a paid job or to start your own business or to get income from farming?**			
	Yes (go to E1_8)	1	No (go to E1_9)	3
**E1_8**	**Where are you looking to find work or employment? (then go to E1_10)** ***[DO NOT read out the options, circle as many as apply]***			
	Asking members of my family	1	Asking for money from banks/micro-finance to start an activity	5
	Asking friends/neighbours	2	Checking advertisements in the newspaper/internet	6
	Asking people I meet in the street/going door-to-door	3	Nothing, I am waiting for someone to offer me a job	7
	Asking for money from family/friends to start an activity	4	Other (please specify)	8
**E1_9**	**Why are you not looking for work or employment? (then go to E1_12)** ***[DO NOT read out the options, circle as many as apply]***			
	I have found a job that I will start soon	1	I don't have the right skills	5
	There are no job opportunities	2	No one is pushing me to find a job	6
	No one offered me a job	3	I don't really need a job	7
	No one wanted to hire a disabled person	4	I have health problems	8
			Other (please specify)<br>____________________	9
**E1_10**	**What kind of work or employment do you have?** ***[DO NOT read out the options, circle as many as apply]***			
	Employed in a fixed job	1	I am a contributing family worker	5
	Employed in occasional work	2	Apprentice	6
	Helping someone with his/her work	3	I have my own business and I have employees	7
	Self-employed	4	Other (please specify)	8

**E1_11**	How satisfied are you with your work/ employment? ***[read the options, circle one option]***	**Not at all satisfied**	**a bit unsatisfied**	**neither satisfied or unsatisfied**	**a bit satisfied**	**completely satisfied**
		1	2	3	4	5

****ONLY ASK E1_12 to E1_15 TO THE HEAD OF HOUSEHOLD. OTHERWISE GO TO E2_1*****

**E1_12**	**What is the household's main source of income? *[DO NOT read the options, circle one option]***			
	No income	1	Money sent home from abroad	7
	Formal paid employment	2	Money sent home from within Liberia	8
	Self-employed	3	Street begging	9
	Family support/donation	4	Government aid	10
	Other individual support (not from family/relatives)	5	Don't know	88
	Aid from international/civil society organisation	6	Other (please specify)	11

**E1_13**	How stable is your household income? ***[read the options, circle one option]***	**Not at all stable**	**A bit unstable**	**Neither stable nor unstable**	**A bit stable**	**Completely stable**
		1	2	3	4	5
					Don't know	88

**E1_14**	**What is the monthly rent? *[write in number]***		(Liberian $)	
**E1_15**	**What is the weekly expenditure of the household? *[write in each number]***			
	Food	L$		-88
	Communication	L$		-88
	Transport	L$		-88
	Other expenditure	L$		-88
	**TOTAL**	**L$**		-88
	Don't know	-88	Refused answer	-99
**E2_1**	**Do you do any chores in the house? *[DO NOT read out the options, circle as many as apply]***			
	No chores	1	Taking care of persons with disabilities in the family	7
	Cooking	2	Laundry/washing clothes	8
	Cleaning	3	Groceries/everyday shopping	9
	Drawing/fetching water	4	Cutting wood	10
	Taking care of elderly family members	5	Other (please specify) ______________________	11
	Taking care of children in the family	6		
**E2_2**	**How many hours per week do you work/do chores? *[write in number, skip if no work AND no chores]***			
	Work	(hours per week)		
	Chores	(hours per week)		

E2_3	Does anyone else do chores around the house ***[read out the options, circle as many as apply]***			
	No	1	Niece/nephew	6
	Parent/grandparent	2	Grandchild	7
	Husband/wife/partner	3	Non-family household member	8
	Son/daughter	4	Household employee	9
	Brother/sister	5	Other (please specify) ___________________	10

TRANSPORT

We would now like to ask you some questions about access to transport

F1_1	How satisfied are you with the access to transport in your community? ***[read the options, circle one option]***	**Not at all satisfied**	**A bit unsatisfied**	**neither satisfied or unsatisfied**	**A bit satisfied**	**completely satisfied**
		1	2	3	4	5

F1_2	What kind of transport do you have access to? ***[read out the options, circle as many as apply]***			
	Own car	1	Own motorbike	7
	Someone else's car	2	Bike	8
	Motorbike taxi (Pempem)	3	Walking	9
	Tricycle / Keke	4	Other (please specify)	10
	Car taxi (collective)	5	Don't know	88
	Bus	6		

F1_3	How often do you have access to the transport you need? ***[read the options, circle one option]***			
	Never (go to F1_4)	1	Most of the time (go to F1_4)	3
	Sometimes (go to F1_4)	2	All of the time (go to G1_1)	4
**F1_4**	**Why don't you have access? *[DO NOT read out the options, circle as many as apply]***			
	Not available	1	Not safe	5
	I don't want to use it	2	Don't know	88
	Costs too much	3	Other (please specify) ________________________	6
	Not disability accessible	4		

SOCIAL INTERACTIONS

Now I would like to ask you some questions about social interactions

G1_1	How included do you feel in your community? ***[read the options, circle one option]***	**Very included**	**A bit included**	**neither included nor not included**	**A bit not included**	**Not included at all**
		1	2	3	4	5

**G1_2**	**Are you a member of any groups or associations?** ***[DO NOT read out the options, circle as many as apply]***			
	No group	0	Parent's and teachers' association (PTA)	8
	Religious institution/group (church or mosque)	1	Other community based/civil society organization	9
	Youth/student's association	2	Market association	10
	Women's association	3	Trade union	11
	Men's association	4	Other professional association	12
	Disabled people's organisation (DPO)	5	Political party	13
	Human rights organisation	6	Other (please specify)	14
	Traditional society	7		
**G1_3**	**Do you participate in any community activities?**			
	Yes (go to G1_4)	1	No (go to G1_5)	2
**G1_4**	**What kinds of community activities? (then go to G1_6)** ***[read out the options, circle as many as apply]***			
	Petty trading	1	Hair dressing	6
	Music	2	Social clubs	7
	Fishing	3	Sports	8
	Community farming	4	Religious activities	9
	Community loan/savings club	5	Other (please specify)	10
**G1_5**	**Why don't you participate in community activities?** ***[DO NOT read out the options, circle as many as apply]***			
	I do not wish to participate	1	I was rejected because a member of my household has a disability	6
	It costs too much money	2	I cannot get around, lack of transport/ inaccessible transport	7
	I think I would be rejected	3	The premises are not disability accessible	8
	I am afraid to do so	4	The attitude of other community members put me off	9
	I tried but I was rejected because of my disability	5	Other (please specify)	10
**G1_6**	**Do you have friends?** ***[read out the options, circle one option]***			
	Yes, many/enough (go to G2_1)	1	No (go to G2_2)	3
	Yes, a few/not enough (go to G2_1)	2		

**G2_1**	How satisfied are you with your relationships with friends	**Not at all satisfied**	**A bit unsatisfied**	**neither satisfied or unsatisfied**	**A bit satisfied**	**completely satisfied**
		1	2	3	4	5
**G2_2**	How satisfied are you with your relationships with your household?	**Not at all satisfied**	**A bit unsatisfied**	**neither satisfied or unsatisfied**	**A bit satisfied**	**completely satisfied**
		1	2	3	4	5
	[only ask if the respondent is in a personal relationship – check question A5_2]					
**G2_3**	How satisfied are you with your relationship with your husband/ wife/partner	**Not at all satisfied**	**A bit unsatisfied**	**neither satisfied or unsatisfied**	**A bit satisfied**	**completely satisfied**
		1	2	3	4	5

	[G3_1 to G3_4 read the options, circle one option]	All the time	Most of the time	Not often	Never
**G3_1**	Do your neighbours help you when you ask for assistance	1	2	3	4
**G3_2**	Do you help your neighbours when they ask for assistance	1	2	3	4
**G3_3**	Does your community help when you ask for assistance?	1	2	3	4
**G3_4**	Do you help your community in community initiatives?	1	2	3	4

**G3_5**	How much do you trust your neighbours?	**Not at all**	**not very much**	**no opinion**	**a bit**	**completely**
		1	2	3	4	5

**G4_1**	**Do you vote? *[read out the options, circle one option]***			
	Yes, always (go to G4_3)	1	No (go to G4_2)	3
	Yes, sometimes (go to G4_2)	2	Refused question (go to G4_3)	99
**G4_2**	**Why don't you vote/always vote? *[DON'T read out the options, circle as many as apply]***			
	I am not registered to vote	1	The polling station is not accessible to me	5
	I don't think it will make a difference	2	I tried to vote but I was turned away	6
	I can't get to the polling station	3	Other (please specify)	7
	I can't read the ballot paper	4	Don't know	88

**G4_3**	How included do you feel in the decision making of your community? ***[read the options, circle one option]***	**Very included**	**A bit included**	**neither included nor not included**	**A bit not included**	**Not included at all**
		1	2	3	4	5

CRIME AND SAFETY

We would now like to ask you some questions about crime and safety

**H1_1**	**In the last year has the level of crime in your community… *[read out the options, circle one option]***			
	Got better	1	Got worse	3
	Stayed the same	2	Don't know	88
**H1_2**	**In the last year has your community been affected by any conflict or dispute?**			
	Yes	1	Don't know	88
	No	2	Refused answer	99
**H1_3**	**Have you personally experienced any form of crime or violence in the last year? *[read out the options, circle one option]***			
	Yes, more than once (go to H1_4)	1	Never experienced crime or violence (go to H1_5)	4
	Yes, once (go to H1_4)	2	Don't know (go to H1_5)	88
	Not in the last year (go to H1_5)	3	Did not answer (go to H1_5)	99

**H1_4**	**What sort of crime or violence?** ***[DO NOT read out the options, circle as many as apply]***			
	Assault, beating or physical injury	1	Child abuse or neglect	8
	Theft or robbery	2	Child trafficking, child sexual exploitation or child prostitution	9
	Sexual abuse/harassment or rape/attempted rape	3	Eviction from the household/community	10
	Sexual exploitation or prostitution	4	Forced labour	11
	Witness murder	5	Don't know/not sure	88
	Intimidation or death threats	6	Refused answer	99
	Ethnic/racial violence or mob violence	7	Other (please specify)	12
**H1_5**	**Has anyone in your household witnessed any crime or violence in the last year?** ***[read out the options, circle one option]***			
	Yes, more than once (go to H1_6)	1	Never witnessed crime or violence (go to H2_1)	4
	Yes, once (go to H1_6)	2	Don’t know (go to H2_1)	88
	Not in the last year (go to H2_1)	3	Did not answer (go to H2_1)	99
**H1_6**	**What sort of crime or violence?** ***[DO NOT read out the options, circle as many as apply]***			
	Assault, beating or physical injury	1	Child abuse or neglect	8
	Theft or robbery	2	Child trafficking, child sexual exploitation or child prostitution	9
	Sexual abuse/harassment or rape/attempted rape	3	Eviction from the household/community	10
	Sexual exploitation or prostitution	4	Forced labour	11
	Witness murder	5	Don't know/not sure	88
	Intimidation or death threats	6	Refused answer	99
	Ethnic/racial violence or mob violence	7	Other (please specify)	12

**H2_1**	**How often do you feel safe in your home?** ***[read out the options, circle one option]***			
	Never	1	Most of the time	3
	Sometimes	2	All of the time	4
**H2_2**	**How often do you feel safe outside of your home?** ***[read out the options, circle one option]***			
	Never	1	Most of the time	3
	Sometimes	2	All of the time	4
**H3_1**	**Do you know where to go if you are victim of crime?**			
	Yes (go to H3_2)	1	No/not sure (go to H4_1)	2
**H3_2**	**Where would you go if you were a victim of crime?** ***[DO NOT read out the options, circle as many as apply]***			
	Go to community leader (e.g. elder, clan chief, town chief)	1	Go to religious leader	9
	Go to justice of peace	2	Go to a disabled people's organisation	10
	Go to police	3	Work with Human Rights organisation	11
	Go to other family members	4	Resort to the use of violence	12
	Go to neighbour or friend	5	Go to trial by ordeal (Sassy Wood)	13
	Go to constituent leaders/ representatives	6	Don't know	88
	Go to district commissioner	7	Did not answer	99
	Go to court	8	Other (please specify)	14

**H4_1**	How satisfied are you with your personal safety? ***[read out the options, circle one option]***	**Not at all satisfied**	**A bit unsatisfied**	**neither satisfied or unsatisfied**	**A bit satisfied**	**completely satisfied**
		1	2	3	4	5
**H4_2**	How satisfied are you with the safety of your household? ***[read out the options, circle one option]***	**Not at all satisfied**	**A bit unsatisfied**	**neither satisfied or unsatisfied**	**A bit satisfied**	**completely satisfied**
		1	2	3	4	5
**H4_3**	How satisfied are you with the safety in your community? ***[read out the options, circle one option]***	1	2	3	4	5

**H4_4**	**Do you think you will feel more safe or less safe 1 year from now?**			
	More safe	1	Less safe	3
	No change	2	Don’t know	88
**H4_5**	**Do you think you will feel more safe or less safe 5 years from now?**			
	More safe	1	Less safe	3
	No change	2	Don’t know	88
**H4_6**	**Do you think that you will be richer or poorer 1 year from now?**			
	Richer	1	Poorer	3
	No change	2	Don’t know	88
**H4_7**	**Do you think that you will be richer or poorer 5 years from now?**			
	Richer	1	Poorer	3
	No change	2	Don’t know	88

We have talked about a lot of different things today. I am now going to give you some cards with cover each of the themes. I would like you to put them in the order that you think they are the most important.

I1

**Rank**	**Card #**
1	
2	
3	
4	
5	
6	
7	
8	
9	

Thank you for taking the time to answer my questions.
